# Critical Flicker Fusion Frequency: A Marker of Cerebral Arousal During Modified Gravitational Conditions Related to Parabolic Flights

**DOI:** 10.3389/fphys.2018.01403

**Published:** 2018-10-02

**Authors:** Costantino Balestra, Marie-Laure Machado, Sigrid Theunissen, Ambre Balestra, Danilo Cialoni, Christian Clot, Stépane Besnard, Laura Kammacher, Julie Delzenne, Peter Germonpré, Pierre Lafère

**Affiliations:** ^1^Environmental, Occupational and Ageing “Integrative Physiology” Laboratory, Haute Ecole Bruxelles-Brabant (HE2B), Brussels, Belgium; ^2^Divers Alert Network (DAN) Europe Research Division, Brussels, Belgium; ^3^UNICAEN, INSERM, COMETE, Normandie Université, Caen, France; ^4^Belgian Road Safety Institute (BRSI), Brussels, Belgium; ^5^Centre for Hyperbaric Oxygen Therapy, Military Hospital “Queen Astrid”, Brussels, Belgium; ^6^Laboratoire ORPHY, EA4324 UFR Sciences et Techniques, Université de Bretagne Occidentale, Brest, France

**Keywords:** 0g, microgravity, brain countermeasures, alertness, space flights, critical flicker fusion frequency, hypergravity, CFFF

## Abstract

*In situ* evaluation of human brain performance and arousal remains challenging during operational circumstances, hence the need for a rapid, reliable and reproducible tool. Here we hypothesized that the Critical flicker fusion frequency (CFFF) reflecting/requiring visual integration, visuo-motor skills and decision-taking process might be a powerful, fast and simple tool in modified gravity environments. Therefore 11 male healthy volunteers were assessed for higher cognitive functions with CFFF during parabolic flights. They were assessed at different time points: upon arrival to the base, 30 min after subcutaneous scopolamine administration, before parabolas, during hypergravity and microgravity at break time (between the 16th and the 17th parabola), on the return flight and on the ground after landing. First, a stable, and consistent measurement of CFFF could be obtained within 12 s. Second, under modified gravitational conditions, the perceptual ability of participants is significantly modified. Compared to the baseline, evolution is characterized by a significant increase of CFFF when in microgravity (0g: 106.9 ± 5.5%), and a significant decrease of CFFF while in hypergravity (2g: 94.5 ± 3.8%). Other time-points were not statistically different from the baseline value. Although the underlying mechanism is still debated, we suggest that the CFFF test is a global marker of cerebral arousal as the result of visuo-motor and decision taking testing based on a simple visual stimulus with an uncomplicated set up that could be used under various environmental conditions. The authors express an opinion that it would be advisable to introduce CFFF measurement during spaceflights as it allows individual longitudinal assessment of individual ability even under conditions of incomplete physiological compensation, as shown here during parabolic flights.

## Introduction

Spaceflight conditions is a complex environmental challenge with multiple consequences for human health. Although physiological consequences have been deeply investigated during the last decades, a large work remains to be performed about cognitive performances and brain adaptation to microgravity conditions (White et al., 2016). For instance, spaceflight can induce maladaptation related to perceptual mismatches between various sensory information, or adverse effects due to physical stressors related to gravitational changes (Strewe et al., 2012). So far, parabolic flights are the best model to reproduce these conditions without traveling to outer space (Montag et al., 2016). During these flights, subjects are exposed to abrupt variation of gravitational conditions causing short high-intensity stresses; 20 s of microgravity (0g) preceded and followed by 20 s of hypergravity (1.8g, roughly 2g) (Pletser et al., 2015). Those severe fast-changing conditions heavily question how the human body and brain react to this unique experience of being weightless and the straining phases of hypergravity. Indeed, operators’ tasks and subjects’ testing, working on-board require cognitive performances while environmental constraint remains high including stress, tiredness, strong cardiovascular stimulation (Negishi et al., 2017), attention and emotional states changes (Collado et al., 2017), risk of motion sickness (Golding et al., 2017). However, the continuous cognitive monitoring all along the flight remains challenging although its evaluation appears crucial particularly during biomedical experiments. Indeed, the alertness and cognitive resources of subjects exposed to selective sensorimotor and/or emotional tasks during biomedical protocols may influence their skills and evaluation so that the challenge of measuring cognition through standardized, fast and reliable tests remains highly requested. Moreover, neurophysiological networks supporting the cortical arousal combining central integration of visual inputs, visuo-motor output and decision-taking is still weakly explored according to gravity levels (Schneider et al., 2009; Marusic et al., 2014). Available studies suggest that no or only slight impairments of elementary and complex cognitive functions or spatial processing were found in space, clear disturbances could be identified in visuo-motor tracking and dual-task performance (Eddy et al., 1998; Manzey and Lorenz, 1998). It also seems that exposure duration is critical as the first 3 weeks of long-term spaceflights and the first 2 weeks back on Earth represent critical periods where adverse effects on attentional processes are to be expected (Manzey et al., 1998). However, results from a 2014 published review by NASA emphasized that due to inconsistent methods, it remains unclear how spaceflight is associated with cognitive dysfunction (Strangman et al., 2014). Moreover, the highly dynamic nature of parabolic flight likely induces somewhat different cognitive decrements than actual spaceflights or ground-based analogs. Therefore, the need for a tool that can possibly reflect the “cerebral state or arousal” and “cognitive ability” in a very short time (within 20 s) is then of interest in extreme environment.

Since cortical arousal means activation of the reticular formation of the brain and as a consequence, an increased wakefulness and vigilance, the use of the critical flicker fusion frequency (CFFF) test may address this need. Historically, a correlation between change in CFFF and the mental state (Seki and Hugon, 1976), and electro-encephalogram (EEG) has been reported (Bennett and Cross, 1960) in the extreme environment of deep diving. EEG reflects a range of complex brain activities, therefore CFFF, which appears to correlate with EEG, might usefully reflect changes in brain performance (Berka et al., 2007). This kind of correlation has been recently further proposed among a world class chess player (Fuentes et al., 2018). In this study evaluation of cortical arousal by both CFFF and EEG by the theta Fz/alpha Pz ratio revealed a parallel increase of CFFF fusion threshold and theta Fz/alpha Pz ratio. The results of another recent study also suggested that CFFF and attentional performance are related with a tight relation between the CFFF and occipital gamma band activity both in frequency and power (Kahlbrock et al., 2012).

Our group has already extensively used a simple device in a range of extreme environments to assess inert gas narcosis during SCUBA diving (Lafere et al., 2016; Germonpre et al., 2017). We have demonstrated that the CFFF test provides an assessment of cognitive function that is similar to psychometric testing (inverse correlation between CFFF and math processing or trail-making tasks with a Pearson r of -0.9695 and -0.8731 or -0.90 and -0.86, respectively, in normobaric and hyperbaric conditions, *p* < 0.001) under normobaric (Hemelryck et al., 2013) and hyperbaric conditions (Lafere et al., 2014). Hyperbaric exposure induces nitrogen accumulation within the human body and is responsible for a neurologic syndrome, often compared to alcohol intoxication, called nitrogen narcosis, which is characterized by an impaired cerebral performance/arousal. The results of these different studies confirmed the gradual deterioration of cognitive performance. We can conclude that a decreased cognitive performance is associated with a decreased CFFF, while an increased CFFF is associated with an improved cerebral performance/arousal.

Outside of the field of diving, despite some limitations, several authors have emphasized the advantages of CFFF assessment as a simple quantitative method for measuring alertness and arousal in humans. Indeed, results of CFFF studies realized in this context, show either no change of CFFF measurement (O’Neal et al., 1992) or parallel evolution of CFFF threshold and alertness/arousal (Davranche and Pichon, 2005; Luczak and Sobolewski, 2005; Railton et al., 2009; Sharma et al., 2011).

More specifically, in the field of gravity/environmental studies, it has been advocated to introduce CFFF measurement into pilot’s individual assessment under conditions of incomplete physiological compensation (Truszczynski et al., 2009). More recently, it was also demonstrated that the value of CFFF, measured before and after centrifugation, corresponded to the level of central nervous system (CNS) arousal caused by exposure to + Gz acceleration (Biernacki et al., 2017), an important part of training for pilots, cosmonauts, and astronauts as it enhances tolerance for hypergravity (Biernacki et al., 2012).

The difficulty faced when measuring brain performance have already been evoked during parabolic flights (Marusic et al., 2014) and other authors rose other limitations on physiological measurements (Migeotte et al., 2002; Karmali and Shelhamer, 2008). Consequently, the purposes of the present study were to assess brain performance during parabolic flight conditions using CFFF since behavioral (psychometric) tests are longer to perform than the short sequences of hypergravity and microgravity. In case the CFFF is efficient in detecting rapid changes to hypothesize that the CFFF may be used for longitudinal studies on cortical arousal in humans.

## Materials and Methods

Experimental procedures, conducted in accordance with the Declaration of Helsinki (World Medical, 2013), were approved by the French Ethical Committee (CPP: 2016-13; N° ID RCB: 2015-A02014-45)

**FIGURE 1:**
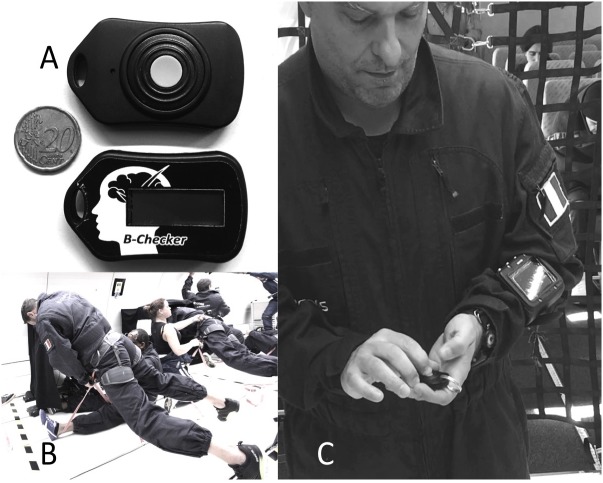
**(A)** The B-Checker device, note the LED position just above the gray button (little hole), it is located at the opposite side of the display, hence the operator is not aware of the result before completing the test. **(B,C)** First author (CB) testing the device during the microgravity and hypergravity phase; the free-floating phase is performed while securely fasten to the ground by means of a rappel harness, the participant faced the ground with no direct light directed to the flickering led nor on the participant eyes.

All methods and potential risks were explained to 12 healthy male volunteers between 34 and 67 years old (48.92 ± 8.75 years) who gave their written informed consent prior to the experiment, which includes the publication of images (as in **Figure 1**). Prior to inclusion into the study, they were assessed fit to fly as they received their medical fitness for parabolic flights. All participants were naïve to the experienced microgravity.

In the present study, CFFF was assessed with a specific device (B-Checker device, I-PHY Company, Braine l’Alleud, Belgium). The design of the device had to consider a number of technical constraints. Indeed, six parameters determine the ability to detect flicker fusion: the intensity of ambient light, the flicker frequency modulation (by convention, a sinusoidal function), the amplitude of the modulation, the average intensity of the illumination, the wavelength or color of the LED, and the position of the stimulus on the retina (Bobon et al., 1982). For instance, at constant brightness, a short flicker is detected at a lower frequency than a long flicker. However, the threshold also varies with brightness, it is higher when the light source is brighter (Feshchenko et al., 1994). Therefore, like all psychophysical thresholds, the flicker fusion threshold is a relative rather than an absolute quantity defined as the frequency at which flicker is detected in 50% of the tests. Depending of the retinal cell type, the absolute value of CFFF frequency (plateau corresponding to the maximum temporal resolution) is set between 15 Hz (rods) and 60 Hz (cones), provided that ambient lighting conditions are sufficient (Hecht and Shlaer, 1936).

Therefore the device was engineered to reach fusion at a lower value of CFFF, to limit the angulation effect and stimulus area (Rovamo et al., 2000) and to blind subjects, who were not aware of the starting and actual flicker frequency before, during and after the test. It consists of a little “key ring like” one button operated system of 5 cm length with an LCD frequency indicator. On the other side of the device a single blue LED (Light Emitting Diode, color temperature 8000 K) (see **Figure 1A**). During the test the subject is looking straight at the LED light at a distance individually adapted to focus both eyes and free from accommodation (generally around 50 cm). Flickering frequency is continuously increased up to fusion. When the subject sees this change in LED light from flicker to fusion, the subject presses the button to stop the test and the reached frequency is recorded. The choice of rising series only instead of alternating rising and falling, a method wildly accepted to circumvent anticipation, was imposed by the time constraint related to parabolic flight (20 s of microgravity sandwiched by 20 s of hypergravity). This choice might be considered as a limitation. However, to mitigate the effect of this decision, steps of increase were made at 0.25 Hz and the final result was automatically rounded up to the higher or lower Hz according to the measure.

The day before parabolic flights, all subjects met the study team in order to familiarize them with procedures and hardware that will be used during the experiment. At this moment, each subject performed the CFFF tests several times before the flight until a consistent result was reached (learning phase) usually 3–5 times, then the actual testing could start. Indeed, to limit the inter- and intra-individual variability of CFFF measurement, it is absolutely essential that the tested subject have a good understanding of when to report the fusion. Based on our previous studies (Hemelryck et al., 2013; Lafere et al., 2016; Germonpre et al., 2017) the CFFF measure does not seem to improve because of learning once understood as confirmed by the reproducibility of basic measurements before each dive in the same individual.

Parabolic flights were programmed on the following Tuesday, Wednesday, and Thursday. Each subject only flew once. One hour before take-off, the subjects received subcutaneous premedication with an anticholinergic (0.175 mg of scopolamine) for the prevention of motion sickness. Scopolamine is a non-selective muscarinic antagonist, producing peripheral antimuscarinic properties and central sedative, antiemetic, and amnestic effects, with a maximum drug concentration occurring approximately 30 min after administration and a duration of action of about 4 h covering the whole procedure (Renner et al., 2005). Since it was not possible to monitor pupil diameter, any ocular side effect could not be excluded. However, since each subject was his own control and, post-medication CFFF measurement was used as baseline measurement, the probability of any bias related to the premedication was limited.

The subjects were assessed for cortical arousal with CFFF at least 4 times at different time points: upon arrival to the base, 30 min after medication, at break time (between the 16th and the 17th parabola), on the return flight, on the ground after landing. Because of other experimental parallel protocols, it was not possible to test each individual during all parabolas (31 parabolas in total). However, participants were tested multiple times during flight (four times between the 1st and 16th parabolas and four times between the 17th and the 31th parabolas): before parabolas, during hypergravity and microgravity. All measurements were performed in a pre-determined area of the Novespace’s aircraft (ZERO-G Airbus A310). During the period of microgravity all subjects were in free floating secured by a strap or harness (see **Figure 1B**). The plane ambient temperature was maintained at 23.4 ± 2.37°C.

### Statistics

All data passed the Kolmogorov–Smirnov allowing us to assume a Gaussian distribution. The intraclass correlation coefficient to test retest reliability for four number of repetitions was calculated at 0.67 indicating a good similarity among the scores. In order to obtain a unique set of measures, for each time point (Preflight, post-medication, during different phases of the parabola [hypergravity (1.8g) and microgravity (0g)], break time, post-parabolas and ground after landing). We calculated for each participant the mean value of CFFF from the available measurements (up to four per time point and participant).

Taking the post-medication value as 100%, percentage changes were calculated, allowing an appreciation of the magnitude of CFFF change between each measurement rather than the absolute values. They were analyzed by means of one-way repeated measures ANOVA and *post hoc* Bonferroni’s test for multiple comparisons. All tests were performed using a standard computer statistical package, GraphPad Prism version 7.00 for Mac (GraphPad Software, San Diego, CA, United States). A threshold of *P* < 0.05 was considered statistically significant. All data are presented as mean ± standard deviation (SD).

## Results

The mean age of the 12 subjects was 48.92 ± 8.75 years; BMI 25.32 ± 2.18 kg/m^2^; weight 80.37 ± 11.21 kg and height 1.779 ± 0.087 m. Although 12 subjects volunteered for the study, the number of complete datasets was reduced to 11 since one subject dropped out due to severe nausea and vomiting.

During the learning phase and the preflight ground phase, subjects were timed. The time to achieve the test was 12 ± 2 s with an intraclass correlation coefficient calculated at 0.72.

Although known side effects of scopolamine such as photophobia and blurred vision, consequences of mydriasis, or even cycloplegia may occur, this seems not to be the case in our study. Indeed, CFFF measurement made 30 min after scopolamine administration (28.8 ± 2.4 Hz) was not statistically different compared to pre-flight (28.9 ± 2.9 Hz) or pre-parabolas (27.6 ± 2.5 Hz) values (one sample *t*-test *p* > 0.05). Nonetheless, we kept the post-medication value as baseline to avoid any bias on further analysis.

**FIGURE 2:**
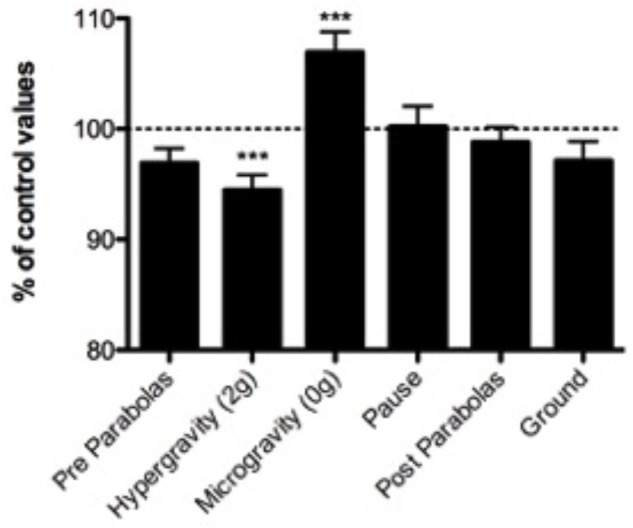
Percentage variation of CFFF at different time points. The post-scopolamine value performed 30 min after subcutaneous injection is taken as 100%. Each subject is compared to his own post-medication value. (^∗∗∗^*p* < 0.001; ns, not significant) (*n* = 11; mean ± SD).

The evolution of CFFF is illustrated in **Figure 2**. Compared to the baseline, evolution is characterized by a significant increase of CFFF during microgravity [0g: 106.9 ± 5.5%; *p* < 0.001 one-way ANOVA, *F*(5,43) = 1.919], and a significant decrease of CFFF during hypergravity [1.8g: 94.5 ± 3.8%; *p* < 0.001 one-way ANOVA, *F*(5,43) = 1.919]. Other time-points (Pre-parabolas: 97 ± 2.0%; Pause: 100.4 ± 4.7%; Post-Parabolas: 99.3 ± 2.4% and Ground 97 ± 5.7%) were not statistically different from the baseline value (*p* > 0.05).

## Discussion

Our cognitive system has adapted to support goal-directed behavior within a normal environment. An abnormal environment, including rapid environmental changes/stressors (e.g., high altitude, cold, microgravity, hyperbaria, etc.), is defined as one to which we are not optimally adapted but can accommodate through the development of coping strategies. However, these coping strategies, especially considering rapid changes in brain performance, are poorly understood.

Ideally, any measurement tool should be reproducible, non-investigator-dependent, designed to observe a change in neurological parameters like EEG measurement, but also easy to implement in hostile or extreme environments. Studying such changes during parabolic flights adds additional constraints. Indeed, the period during which a measurement is possible is limited to 20 s. This requires the use of a very responsive and quick measurement tool. When adding up all the needed specifications this tool does not seem to exist unless we consider the use of CFFF. Indeed, it has to be seen as a global and integrative indicator of brain performance (Lafere et al., 2014, 2016; Germonpre et al., 2017).

A good example is provided in the field of SCUBA diving, also an extreme environment. Studies in this particular field have produced consistent results, that are in accordance with the actual neurochemical theory of nitrogen narcosis (Rostain et al., 2011). Those studies suggested that the net effect on cerebral performance (as measured by CFFF) depends on a balance between the activating effects of oxygen and the inhibitory effects of nitrogen on synthesis, release and recapture of neurotransmitter such as glutamate, dopamine or γ-aminobutyric acid (GABA).

Although such comparisons can be misleading, recent data among airplane pilot trainee, suggest some similarity in the processes involved (Yildiz et al., 2014). Results obtained by Magnetic Resonance Spectroscopy (MRS) demonstrated that higher striatal concentrations of GABA and glutamate/glutamine were related to a superior performance in action control allowing to differentiate between high and normal performer. Since MRS is highly unpractical during parabolic flights, this is another rational to test alternative methods. With this is mind, here we showed that we reached a stable, and consistent measurement within around 12 s of time, which is quick enough for the purpose of parabolic flights. However, since CFFF measurement are influenced by many factors, no conclusions can be made on absolute values. Therefore, we are more interested in analyzing the magnitude of the change. This implies that ambient light stays similar through the whole procedure, which was the case in the present setting [at around 9000 lux measured on the ground and in the plane with an application on a smartphone (Lux Light Meter Free)]. This also implies that this method, in its present form, is only suitable for paired studies where each candidate is his own control. This approach is consistent with the integrative paradigm, who views any systems, as a whole, as providing the more relevant signal (Grocott, 2013), especially when it comes to adaptive mechanisms.

One explanation of our results might be tempted through the function of the retina. Indeed, flicker perimetry is a known alternative for assessment of the magnocellular visual pathways. It has been shown to be useful for the detection of glaucoma and deficits due to ocular hypertension (McKendrick, 2005). However, none of our candidates suffered from these pathologies since they received their medical fitness checking for the lack of such ophthalmologic disease. Several other factors might then be involved. For instance, gravity alterations may be related to undetected changes in pupil diameter, since we did not measure this particular parameter. Indeed, scopolamine is a known factor of pupil size modification. Makoswky et al found a statistically significant changes in pupil size and near point accommodation among parabolic flyers. However, this did not appear to be clinically important as no significant decrement in acuity was noted (Makowski et al., 2011). It is also known that pupils constrict during hypergravity (Cheung and Hofer, 2003), while mydriasis is associated with an increased CFFF threshold (Schmitt et al., 2002). Since we observed an opposite result, i.e., reduced CFFF in hypergravity, other explanations need to be considered. For instance, the blood flow increase in the choroidal system of eye during parabolic flights has been well studied and shows an increase of blood flow during the 0g phase, this may be considered as a confounding factor (Ansari et al., 2003; Taibbi et al., 2013). However, we believe that CFFF is more dependent on the cortical arousal and thus brain blood flow or metabolism as we observed similar changes in CFFF when breathing pure oxygen (Balestra et al., 2012; Hemelryck et al., 2013). Indeed, it is known that oxygen will cause vasoconstriction in the eyes blood flow and in the brain. It has to be noted that in the absence of hemodynamics variation, CFFF measurements parallel brain activation and inhibition assessed by continuous recording of brain near infrared spectroscopy under different partial pressure of oxygen (Lafere et al., 2014). Although the eye is considered a window to the body (Ansari and Sebag, 2008), we nevertheless conclude that the observed changes are more dependent on brain physiology. Indeed, it has been demonstrated that flickering and fused percepts were associated with distinct patterns of activation in response to physically identical flickering stimuli. Specifically, perception of flicker was associated with greater activation in bilateral frontal and left parietal cortex. These findings indicate that activity of higher-level cortical areas is important for awareness of temporally distinct visual events in the context of a non-spatial task (Carmel et al., 2006). It has to be reminded here that another study, exploring the acute effect of microgravity during parabolic flight, demonstrated that complete removal of gravity did not pathologically elevate intracranial pressure (ICP) but did prevent the normal lowering of ICP when upright (Lawley et al., 2017). However, to date, it is difficult to evaluate this effect on CFFF threshold.

According to the available literature on CFFF, as described in the introduction, a decreased CFFF is associated with a deteriorated cerebral performance/arousal, while an increased CFFF is associated with the opposite. Therefore, although cerebral performance was not specifically measured (20 s available to perform cerebral evaluation), the second main finding, based on CFFF threshold, is an improved performance while in microgravity and the opposite, a decreased performance while in hypergravity. We can also consider that an improved arousal/alertness may be a surrogate of an improved performance in some tasks. This slightly differs from previous publications (Manzey and Lorenz, 1998; Jungling et al., 2002; Bock et al., 2003, 2010). In these studies, cerebral performance was evaluated in conjunction with motor control. The allocation of additional resources to motor control may have led to a shortage of resources for other task components, such as display monitoring and decision making, i.e., cerebral performance. In simple words, by limiting the number of synapses involved in a certain task, even if the conduction between neurons is modified, this will not be noticeable if the number of synaptic connections involved is too small. Alternatively, if many neural connections are involved, the integration over the whole path will yield a proportional change in time for the test, as shown by CFFF. Since no complex cognitive task was involved in our design, only the attention was tested. This could explain our results.

Due to the experimental setting, we propose some hypotheses to explain our results. First, it has been suggested that, among astronauts, microgravity may induce changes in neuronal metabolism and architecture of the motor cortex (Cassady et al., 2016; Koppelmans et al., 2016). According to animal studies, the effects of prolonged simulated microgravity elicited a decreased content of GABA and increased the content of glutamate in the rat hippocampus, a vital brain region involved in learning, memory, and navigation (Wang et al., 2015, 2016) and also decreased the expression of crucial genes involved in dopamine synthesis and degradation, as well as the D1 dopamine receptor (Popova et al., 2015). Although tempting, the hypothesis of a neurobiochemical-behavioral coupling cannot be affirmed in the present setting because the kinetic of these changes are currently not known. Unlike pressure/immersion exposure where neurotransmitter response only takes seconds, these coping strategies, while exposed to microgravity or hypergravity, have only been measured in the long run over days to months. Also, it has to be reminded that our subjects were naïve to such exposure and no prior acclimation could happened. Then, it would be interesting to perform the same tests on the safety crewmembers in order to detect a possible adaptive mechanism. Also, performing this test in sustained 0g environment would permit to validate formally the CFFF as a tool to longitudinally evaluate cognitive cerebral function during spaceflight.

Second, changes in gravity are associated with changes in activity of the adrenomedullary, sympathoneural and hypothalamo-pituitary-adrenocortical systems, with increased plasma catecholamine and corticosterone levels. Since, CFFF is sensitive to variations of arterial blood pressure (Gutherie and Hammond, 2004), this might also be an explanation of the observed results. However, again the kinetic of the reaction is not favorable as in available animal models, rats were centrifuged for 10 min up to 6g and catecholamine level followed for 1 h after centrifugation (Petrak et al., 2008) while the stressor in parabolic flights is only 1.6–1.8g for 20 s.

Finally, and most probably, a possible link between cardiovascular effects, cranial volume regulation and CFFF needs to be considered. Indeed, the primary driver of venous blood return is pressure. Venous blood return from the head, neck and upper trunk is typically assisted by gravity, while the venous blood return from the lower half of the body needs the propulsive force of both valves and muscular contraction to oppose gravity. Therefore, some orthostatic difference of cerebral blood flow may also explain the difference between the increased vigilance/arousal during microgravity and reduced performance during hypergravity (Migeotte et al., 2002). Increased blood flow during neural stimulation to fulfill the metabolic demands of the tissue is a well-known mechanism. This neurovascular coupling exists in the retina as well. Therefore, cerebral and retinal blood flow are dependent on local neuronal activity. According to Palkovits et al., applying a flickering light as retinal stimulation increased retinal blood flow by more than 50% and oxygen extraction by more than 30% (Palkovits et al., 2015). This study also demonstrated that during systemic hyperoxia (100% oxygen breathing), the blood flow and oxygen extraction responses to neural stimulation are increased. The inverse relation seems also to be true as we demonstrated that a variation of the oxygen partial pressure influences the CFFF threshold at least in the absence of hemodynamic variations (Lafere et al., 2014). However, it is known that spaceflights lead to a redistribution of venous blood volume, a well-known phenomenon among astronauts, leading when prolonged, to the “fat face, chicken legs” syndrome due to a global fluid shift including interstitial fluid as well (Blomqvist et al., 1994). This fluid redistribution has consequences. For instance, Taibi et al. (2017) showed interesting differences in the amplitude of cardiac oscillations measured at the level of the neck veins, while in a model of ground-based simulated microgravity, Kermorgant et al. (2017) demonstrated that weightlessness could improve cerebral autoregulation. All these changes could be considered as confounding factors, however, these changes have been objectivated after long duration exposure from days to months. Since we did not assess hemodynamics variables, we can only suggest some hypothesis. Nonetheless, it could be possible that a similar mechanism is involved in acute exposures as in parabolic flight. Therefore, the increase of CFFF during free floating phases (microgravity) could probably be due to an increase of cerebral blood flow. This hypothesis is consistent with infrared images of facial skin temperature obtained during various phases of a parabolic flight. The rapid dynamics of skin temperature changes that are indeed caused by a fluid shift along the body axis and during weightlessness lead to a striking increase in heat emission (Gunga et al., 2009). It is also possible that a differential parasympathetic nervous system modulation, as demonstrated among astronauts could also explain our results (Sigaudo-Roussel et al., 2002). In any cases, more research is needed to better understand the underlying mechanism.

## Conclusion

Although the underlying mechanism is still debated, we conclude, as previously demonstrated by our group, that the CFFF test provides a global assessment of cortical arousal with an uncomplicated set up that could be used under various environmental conditions. Using CFFF, it would thus be possible to conveniently estimate cognitive abilities rapidly. Further researches are then needed in order to correlate the CFFF variations with complex cognitive tasks and its possible adaptive effects performed in such altered gravity conditions. Since there is ample evidence of the nonlinear (inverted U shape) relationship between arousal and cognitive performance (Yerkes–Dodson Law; Gur et al., 1988), CFFF should also be tested in its capacity to predict declining performance at higher than optimal arousal levels.

## Author Contributions

CB, SB, ST, M-LM, PG, and PL conceived and planned the experiments. CB, SB, M-LM, CC, and LK carried out the experiments. LK, AB, and DC planned and carried out the simulations. CB, ST, AB, DC, PG, JD, and PL contributed to the interpretation of the results. PL took the lead to write the manuscript. All authors provided critical feedback and helped shape the research, analysis, and the manuscript.

## Conflict of Interest Statement

The authors declare that the research was conducted in the absence of any commercial or financial relationships that could be construed as a potential conflict of interest.
